# Association between the *PTPN22 *+1858 C/T polymorphism and psoriatic arthritis

**DOI:** 10.1186/ar3284

**Published:** 2011-03-16

**Authors:** Kristina Juneblad, Martin Johansson, Solbritt Rantapää-Dahlqvist, Gerd-Marie Alenius

**Affiliations:** 1 Department of Public Health and Clinical Medicine, Rheumatology, University Hospital, SE-901 85 Umeå, Sweden

## Abstract

**Introduction:**

The purpose of the present study was to investigate the frequency of the *PTPN22 *+1858 C/T single nucleotide polymorphism (SNP) (rs 2476601), previously shown to be associated with several autoimmune diseases, in patients with psoriatic arthritis (PsA) in comparison with population based controls.

**Methods:**

A total of 291 patients (145 male/146 female, mean age (± S.D.) 52.2 (± 13.1) years) with PsA were examined clinically, by standard laboratory tests and their DNA was genotyped for the SNP rs2476601 (*PTPN22 *+1858 C/T). Allelic frequencies were determined and compared with 725 controls.

**Results:**

Carriage of the risk allele, *PTPN22*+1858T, showed a significant association with patients with PsA compared with controls (χ^2 ^= 6.56, *P *= 0.010, odds ratio (OR) 1.49; 95% confidence interval (CI) 1.10 to 2.02). A significantly higher proportion of carriers of the risk allele (T) had significantly more deformed joints (n ± SEM) (5.9 ± 1.2 vs 2.8 ± 0.5; *P *= 0.005).

**Conclusions:**

In this study the +1858T allele of the *PTPN22 *gene, known to be associated with several autoimmune diseases, was associated with PsA. The finding of significantly more joints with deformities among carriers of the T variant could indicate a more aggressive phenotype of disease.

## Introduction

Psoriatic arthritis (PsA) is a heterogeneous inflammatory arthritis associated with psoriasis. The disease severity not only varies between patients but also within an individual patient over time. The disease expression can vary from a mild mono-oligoarthritis to severe erosive polyarthritis comparable with rheumatoid arthritis (RA) [1]. In contrast to RA, manifestations such as dactylitis and enthesitis are common in patients with PsA, as is the case in patients suffering other diseases within the sero-negative spondylarthropathy group [2,3]. Also, in contrast with RA, most individuals with PsA are sero-negative for rheumatoid factor (RF) and anti-citrullinated protein/peptide antibodies (ACPA) [4,5].

As with many other autoimmune diseases a number of genes have been suggested to be associated with PsA [6,7]. Epidemiological data implicate a strong genetic basis for PsA [6,8]. Familial aggregation with an estimated recurrence risk ratio in first degree relatives (λ_1_) of 55 in different studies compared with 5 to 10 for patients with cutaneous psoriasis, has been reported in different studies [6,8]. Previous genetic studies have shown multiple polymorphisms within the MHC region on chromosome 6p to be associated with PsA together with a number of candidate genes outside this region being suggested [6,7].

The protein tyrosine phosphatase non-receptor 22 (*PTPN22) *gene, located on chromosome 1p13, codes for a protein, Lyp, thought to function as a negative regulator of T-cells [9] although a role in B-cell signaling has also been recently suggested [10]. The single nucleotide polymorphism (SNP) rs2476601 (+1858C/T), located in exon 14 of the *PTPN22 *gene, has previously been found associated with several autoimmune diseases, for example, diabetes type-I [11] and RA, with a stronger association with ACPA sero-positive RA [12,13]. Previous studies investigating an association between *PTPN22 *+1858C/T and susceptibility to PsA have shown conflicting results [14-16].

The aim of the present study was to ascertain whether the *PTPN22 *+1858C/T polymorphism was associated with susceptibility to, or severity of, disease in well-characterized patients with PsA from northern Sweden.

## Materials and methods

### Patients

This case-control study comprised 291 consecutively included patients with PsA (145 male/146 female, with a mean age (± S.D.) of 52.2 (± 13.1) years) and 725 controls (265 male/460 female, mean age (± S.D.) 55.6 (± 12.4). All patients and controls were from the same geographic area of northern Sweden and all controls were randomly selected from the Medical Biobank of Northern Sweden. PsA was diagnosed when a patient presented with an actual psoriasis, or a history of psoriasis, of the skin combined with inflammatory arthropathy defined as peripheral arthritis (out of 66/68 joint) of more than six weeks duration and/or radiologically assessed axial involvement based on radiological findings in the sacroiliac joints according to the New York criteria (≥2) [17] and/or syndesmophytes, ligamentous ossification, vertebral squaring and shining corners of the spine [18]. Dactylitis was defined as painful swelling and inflammation of a finger or a toe and, deformed joints were defined as radiological erosions and/or irreversible deformations (for example, ankylosis, subluxation and/or loss of function or reduced mobility). Patients were examined clinically, by laboratory based analysis and, if needed for proper classification of diagnosis, radiologically, and. subsequently, classified according to the criteria of Moll and Wright and of the CASPAR study group [1,5]. The local ethics committee at Umeå University, Sweden, approved the study, and all patients and controls gave their written informed consent. Table 1 shows the demographic data and phenotype of the patients at the time of the study.

**Table 1:** Demographic data and phenotype of patients at the time of the study

Age (years)	52.2 ± 13.1
PsA duration (years)	15.2 ± 11.7
Tender joints (mean ± SEM)	6.6 ± 0.5
Swollen joint (mean ± SEM)	4.4 ± 0.3
Duration of skin disease (years)	25.3 ± 14.8
Duration of joint disease (years)	14.3 ± 11.4
ESR mm/h	16.2 ± 15.7
CRP mg/L	10.5 ± 8.1
Arthritic joints (mean ± SEM)	3.0 ± 0.2
Deformed joint (mean ± SEM)	3.8 ± 0.5
	
Rheumatoid factor positive	34 (11.9%)
Anti-citrullinated protein/peptide antibodies positive	21 (7.3%)
Nail involvement	121 (42.6%)
DIP-joint involvement	93 (33.2%)
Dactylitis "ever"	64 (23.4%)
Fulfilling CASPAR	247 (92.9%)
Mono-/oligoarthritis	117 (41.3%)
Polyarthritis	135 (47.7%)
Axial involvement	60 (20.8%)

Data presented by mean ± SD or n (%) when appropriate, unless stated otherwise.

### Genotyping

DNA from the patients and controls was extracted from ethylenediamine tetraacetic acid-treated whole blood using a standard technique and genotyped for the SNP rs2476601 (*PTPN22 *+1865C/T) allele by the TaqMan^® ^assay using an ABI PRISM 7900HT Sequence Detection System and the SDS 2.1 software (Applied Biosystems, Foster City, CA, USA) according to the manufacturer's instructions.

### Laboratory analysis

Blood samples were collected when the patients were assessed clinically. The erythrocyte sedimentation rate (ESR; mm/h) and C-reactive protein (CRP; mg/L) were analyzed using standard protocols by the authorized chemical laboratory at the University Hospital of Umeå. Rheumatoid factor (RF) was measured using the ORG 522 M Rheumatoid Factor IgM ELISA kit (Orgentic Diagnostika GmbH, Mainz, Germany) with a cut-off value >20 IU/mL. The presence of anti-citrullinated protein/peptide antibodies (ACPA) was determined using the DIASTAT Anti-CCP ELISA kit FCCP200 from Axis-Shield Diagnostics Limited (Technology Park, Dundee, Scotland, UK) and a cut off value >5 U/mL.

### Statistics

Statistical calculations were performed using SPSS 16.0 (SPSS Inc., Chicago, IL, USA). The Chi-square test was used for testing categorical data between groups. Odds ratio (OR) was calculated with 95% confidence interval (CI). All *P*-values refer to a two-sided test and a *P*-value ≤0.05 was considered statistically significant. For multivariate analysis, logistic regression analysis was used. Power calculation was performed using EpiInfo. To estimate the number of patients and controls needed, frequency data from a previous study were used [16].

## Results

The genotype and allele distribution of the *PTPN22 *+1858C/T SNP among patients and controls were in agreement with the Hardy-Weinberg equilibrium. Using the minor allele frequency of 0.085, which was reported for the Toronto population in an article by Butt *et al. *[16], the present study had more than 80% power to detect an effect size of 2.00 and a power near 70% to detect an effect size of 1.8 and approximately 35% power to detect an effect size of 1.5.

Carriage of the *PTPN22 *+1858T variant (CT + TT) was significantly increased in patients with PsA compared with controls (χ^2 ^= 6.56, *P *= 0.010, OR 1.49 (95% CI 1.10 to 2.02)) (Table 2). Allelic frequency of risk allele, T, was also significantly increased in the PsA patients, that is, 0.160 versus 0.119. The association was further increased when RF sero-positive patients were excluded (χ^2 ^= 8.56, *P *= 0.003, OR 1.61 (95% CI 1.17 to 2.21)) as well as when ACPA sero-positive patients were excluded (χ^2 ^= 6.63, *P *= 0.010, OR 1.51 (95% CI 1.10 to 2.07)). Only 6 patients (2.1%) and 10 controls (1.3%) were homozygous for the T-allele (χ^2 ^= 0.62, *P *= 0.430). This subgroup is thus too small to allow appropriate statistical calculations.

**Table 2:** Frequency distribution of *PTPN22 *+1858 C/T polymorphism in psoriatic arthritis patients and controls

Genotype	Patients (*n *= 291) n (%)	Controls (*n *= 725) n (%)	χ^2^	*P*-value	OR	95% CI
**CC**	204 (70.1)	563 (77.7)	6.40	0.011	0.67	0.49 to 0.93
**CT**	81 (27.8)	152 (21.0)	5.54	0.019	1.45	1.05 to 2.01
**TT**	6 (2.1)	10 (1.3)	0.62	0.430	1.51	0.48 to 4.54
**CT+TT**	87 (29.9)	162 (22.3)	6.56	0.010	1.49	1.10 to 2.02
**T allele**	93 (16.0)	172 (11.9)	6.21	0.013	1.41	1.07 to 1.87

CI, confidence interval; OR, odds ratio.

When analyzing the separate disease phenotypes, a significantly higher proportion of carriers of the risk allele, T, had, at some point, been diagnosed with dactylitis (χ^2 ^= 10.56, *P *= 0.001, OR 2.58 (95% CI 1.44 to 4.62)). Furthermore, they had significantly more deformed joints at examination compared with non-carriers (mean number of joints ± SEM; 5.9 ± 1.2 vs 2.8 ± 0.5; *P *= 0.005), and this association remained significant after multiple logistic regression analysis (*P *= 0.017). There was no association between dactylitis and deformed joints and, no other significant associations between the carriage of the risk allele, T, and other examined disease phenotypes, for example, number of swollen or tender joints at examination, axial disease, level of ESR or CRP, nail psoriasis or distal interphalangeal (DIP)-joint involvement (data not shown). Furthermore, there was no significant difference in the allelic distribution in patients with mono-oligoarthritic disease compared with those having a polyarthritic disease expression (data not shown).

There were no significant differences in genotyped allelic frequencies between males and females, either for the patients or the controls (data not shown).

## Discussion

In this study of clinically and laboratory well characterized patients with PsA, the +1858T allele of the *PTPN22 *gene, previously shown to be associated with several autoimmune diseases, was found to be associated with a diagnosis of PsA.

Previous association studies regarding *PTPN22 *+1858C/T polymorphism and PsA have produced conflicting results. In a study by Hinks *et al*., no association was found between PsA and *PTPN22 *+1858C/T in a patient population from the United Kingdom [14]. Likewise, no association was found between PsA and *PTPN22 *+1858C/T in a German cohort of 375 patients with PsA when the patient group was considered as a whole; however, a significantly higher proportion of males carried the risk allele [15]. In a study of two Canadian populations, one from Toronto and one from Newfoundland, an association between *PTPN22 *+1858C/T and PsA was only found in the Toronto population [16]. In that study, the T-allele frequency of the Toronto population was significantly increased in PsA patients, 0.138 compared with 0.085 in controls. Thus, our results are consistent with those reported for the Toronto population by Butt *et al*., but are in contrast with the others cited. This could indicate that association with *PTPN22 *exists in specific populations, as to date exemplified by the Toronto population by Butt *et al*. and the population from northern Sweden in the present study. Alternatively, as suggested by the Canadian authors, a false positive association is, of course, possible due to population stratification. It is also possible that further studies with increased sample sizes would detect significant associations among other populations. The association with the male gender observed within the German cohort [15] was not confirmed by this study in which no differences in allelic frequencies between males and females could be detected. In all three studies cited here the distribution between patients and controls are different compared with the present study. The patient:control ratio in our study is 291:725, in the UK cohort 455:595 [14], in the German patient group 375:299 [15], in the Newfoundland cohort 238:149 and in the Toronto cohort 207:199 [16]. These differences make direct comparison between the various studies difficult. However, following our calculations, the only study with more power than the present study is that based on the UK cohort; in all of the other studies the power value is at the same level or lower than in the present study. Since the allelic frequency of *PTPN22*+1858T varies considerably among different populations, for example, within Caucasian populations in Europe it varies from 2 to 3% in the south of Europe to >10% in Scandinavian countries [19] it is important to incorporate an appropriately large group of controls with a homogeneous origin. Our control group was relatively large compared with the previously published studies and was selected from the same geographic area as the patients. A possible explanation for the positive association between *PTPN22 *and PsA could have been that misdiagnosed patients were included in the study. Anyhow, when patients with positive RF and ACPA were excluded, the OR increased, indicating that the result was not caused by misdiagnosed patients.

Interestingly, our study revealed that carriers of the risk allele, *PTPN22 *+1858 T, had significantly more joints with deformities. There was no association found with a polyarthritic disease pattern, thus the results of this study could indicate that carriage of the risk allele, T, is a risk factor for a more aggressive form of PsA, although not with a disease pattern having similarities to RA.

A limitation of this study is that the number of patients studied does not allow proper stratification of the data, for example, the number of RF and ACPA sero-positive subjects is too small to allow appropriate calculation. Therefore, it was not possible to evaluate properly any likely association in these sub-groups.

## Conclusions

In conclusion, this study shows that the +1858T allele in the *PTPN22 *gene, previously shown to be associated with several autoimmune diseases, is also associated with PsA in patients from northern Sweden, a result that is consistent with previous data regarding a population from Toronto. Carriers of the T-allele also had significantly more joints with deformities compared with non-carriers of the T-allele, possibly indicating a more aggressive phenotype of disease.

## Abbreviations

ACPA: anti-citrullinated protein/peptide antibodies; CI: confidence interval; DIP: distal interphalangeal; OR: odds ratio; PsA: psoriatic arthritis; *PTPN22*: protein tyrosine phosphatase non-receptor 22; RA: rheumatoid arthritis; RF: rheumatoid factor; SNP: single nucleotide polymorphism.

## Competing interests

The authors declare that they have no competing interests.

## Authors' contributions

KJ, the main investigator, carried out the genotyping together with MJ, performed laboratory and statistical analysis, and contributed to preparation of the manuscript. GMA is the principal investigator, who, together with SRD, is responsible for the samples from the Biobank, designed the investigation, and participated in the data collection, statistical analysis and drafting of the manuscript.
